# Cyclic fatigue resistance of 2Shape, Twisted File and EndoSequence Xpress nickel-titanium rotary files at intracanal temperature

**DOI:** 10.15171/joddd.2018.044

**Published:** 2018-12-19

**Authors:** Gülşah Uslu, Taha Özyürek, Mustafa Gündoğar, Koray Yılmaz

**Affiliations:** ^1^Department of Endodontics, Faculty of Dentistry, Çanakkale Onkekiz Mart University, Çanakkale, Turkey; ^2^Department of Endodontics, Faculty of Dentistry, Ondokuz Mayıs University, Samsun, Turkey; ^3^Department of Endodontics, Faculty of Dentistry, Medipol University, İstanbul, Turkey; ^4^Department of Endodontics, Faculty of Dentistry, Hatay Mustafa Kemal University, Hatay

**Keywords:** Cyclic fatigue, EndoSequence Xpress, Twisted File, 2Shape, static test

## Abstract

***Background.*** The aim of this study was to compare the cyclic fatigue resistance of 2Shape, Twisted File (TF) and EndoSequence Xpress (ESX) nickel-titanium rotary files at intracanal temperature (35°C).

***Methods.*** Twenty 2Shape TS1 (25/.04), 20 TF (25/.04) and 20 ESX (25/.04) files were tested for cyclic fatigue at intracanal temperature (35°C). All the instruments were rotated in artificial canals which were made of stainless steel with an inner diameter of 1.5 mm, 60° angle of curvature and a radius curvature of 5 mm until fracture occurred; the time to fracture was recorded in seconds using a digital chronometer and the number of cycles to fracture (NCF) for each file was calculated. Kruskal-Wallis test with Bonferroni correction was performed to statistically analyze data using SPSS 21.0. Statistical significance was set at P<0.05.

***Results.*** NCF values revealed that the 2Shape had significantly the highest cyclic fatigue resistance, followed by TF and ESX at intracanal temperature (P<0.05). The difference was significant between the TF and ESX groups (P<0.05). There was no significant difference among the 2Shape, TF and ESX files with respect to the lengths of the fractured file fragments (P>0.05).

***Conclusion.*** Within the limitations of present study, it was concluded that the cyclic fatigue resistance of 2Shape files at the intracanal temperature is higher than that of TF and ESX files.

## Introduction

Despite many advantages of nickel-titanium (NiTi) rotary instruments, their unexpected fracture during the root canal preparation is a source of concern for clinicians.^1^ File fractures that might negatively affect the success of root canal treatment occur due to cyclic or torsional fatigue without giving any prior sign. The cyclic fatigue failures of files occur as a result of accumulation of repetitive compressive and tensional forces that the file is exposed to within the curved canals, whereas the torsional fatigue failures occur when a part or tip of the file is stuck in the canal but its coronal segment continues rotating.^2^ Some of the reasons for file fractures are factors such as root canal anatomy,^3^ design of the files, the features of the alloy used in production^4^ and repetitive use of files.^5^

The asymmetric rotation of EndoSequence Xpress (ESX; Brasseler, Savannah, USA) NiTi rotary file, which was recently introduced to the market, makes it easier to transport the debris to the coronal area due to its triangular cross-sectional area. The manufacturer claims that the electro-polishing applied on the surface of file increases the cyclic fatigue resistance of file. Its patented 6-cutting-edge booster tip structure ensures more efficient and safer shaping, as well as minimizing the formation of intracanal complications.^6^

2Shape (MicroMega, Besancon, France) rotary file system consists of two files as TS1 (25/.04) and TS2 (25/.06). The T-Wire technology used in the production of files is claimed to increase the cyclic fatigue resistance of files by 40% in comparison to One Shape (MicroMega) files. 2Shape has a latest generation of cross-section with triple helix with 2 main cutting edges for cutting efficiency and one secondary edge for improved removal of debris.^7^

Twisted File (TF; Axis/SybronEndo, Orange, CA), which is another file system, is manufactured by twisting the metal alloy using a special heat treatment (R-Phase) in order to increase the cyclic fatigue resistance and super-elasticity of the ile.^8^

To date, many of the studies examining the cyclic fatigue failures of files have been carried out at room temperature.^9-12^ However, since the room temperature is lower than the intracanal temperature (35±1°C),^13^ the tests’ ability to mimic the clinical conditions declines. In a comprehensive literature review performed here, no study examining the cyclic fatigue resistance of 2Shape root canal instrument was found. The aim of the present study was to compare the cyclic fatigue resistance of 2Shape, ESX and TF NiTi rotary files at intracanal temperature (35°C). The null hypothesis was that there would be no difference between the cyclic fatigue resistance of these files.

## Methods

Based on data from a previous study,^14^ a power calculation was performed using G*Power 3.1 (Heinrich Heine University, Dusseldorf, Germany) software. The calculation indicated that the sample size for each group should be a minimum of 20 files. Thus 20 2Shape (25/.04), 20 ESX (25/.04) and 20 TF (25.04) files were included in the present study. Before the cyclic fatigue test, the 60 NiTi files were examined under a stereomicroscope (Olympus BX43; Olympus Co, Tokyo, Japan) at ×20 magnification to ensure that no defects that could lead to deformation were present.

The artificial canal used in the study was made of stainless-steel and manufactured to reproduce the instrument sizes and taper. It had a 60° angle of curvature and a 5-mm radius of curvature.^15^ All the files were operated in 35°C water^16^ using a torque-controlled endodontic motor (VDW Gold; VDW Munich, Germany) following the instructions of use given by the manufacturers: 2Shape at 300 rpm, ESX at 500 rpm and TF at 500 rpm. To be able to define temperatures, a glass container was filled with 300 mL of water at 37°C. The original temperature of the water was 22°C. To achieve the desired temperatures, the glass container was placed on a hot plate until the water temperature was stabilized at 37ºC during all the tests.

The time to fracture was recorded in seconds for each instrument, using a digital chronometer and the number of cycles to fracture (NCF) for each file was calculated using the following formula: (NFC = revolutions per minute (rpm) × time to fracture (sec)/60). A digital microcaliper was used to determine the length of each fractured fragment (FL). The mean FL was recorded to evaluate the correct positioning of the tested instrument inside the canal curvature and to determine whether similar stresses were induced.

To determine the fracture type of the files, two pieces of fractured files from each group (eight pieces in total) were examined under a scanning electron microscope (SEM) (JEOL, JSM-7001F, Tokyo, Japan). Photomicrographs of the fractured surfaces were obtained under different magnifications (×100 to ×3000).

### Statistical analysis

The NCF and FL data were first analyzed using Shapiro-Wilk test to verify the assumption of normality. Kruskal-Wallis test with Bonferroni correction was performed to statistically analyze data using SPSS 21.0 (IBM-SPSS Inc, Chicago, IL). Statistical significance level was set at P<0.05.

## Results

The means and standard deviations of the NCF and FL values of the tested NiTi files are shown in Table 1. NCF values revealed that the 2Shape had significantly the highest cyclic fatigue resistance followed by TF and ESX at intracanal temperature (P<0.05). The difference was significant between the TF and ESX groups (P<0.05). There was no signiﬁcant difference between the 2Shape, TF and ESX ﬁles with respect to the lengths of the fractured ﬁle fragments (P>0.05).

**Table 1:** Mean and Standard Deviations of the Number of Cycles to Failure and the Length of the Fractured Fragment of the tested NiTi files at 35° C.

	**Number of Cycles to Failure**	**Fractured Length (mm)**
**Twisted File**	987.48 ± 121.18 ^a^	5.54 ± 0.35
**ESX**	355.58 ± 68.08^b^	5.68 ± 0.33
**2Shape**	1242.24 ± 192.32 ^c^	5.02 ± 0.41
***P*** **-value**	< 0.05	> 0.5

* Different superscripts indicate statistically significant difference (*P* < .05).

**Figure 1:**
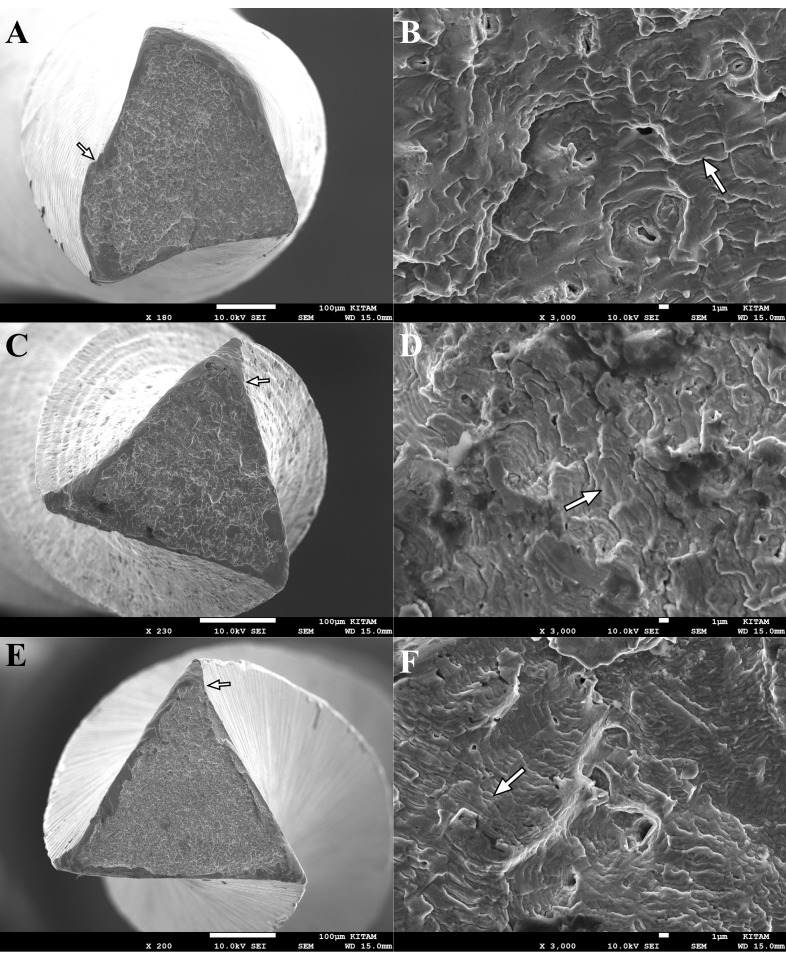
Scanning electron microscopic appearances of the 2Shape, ESX, and TF files after cyclic fatigue testing. The fracture surface view of (A) 2Shape, (C) ESX, and (E) TF and a high-magnification view of (B) 2Shape, (D) ESX, and (F) TF instruments. The crack initiation origin and the surface pattern with dimples and cones are observed in the same fracture surface (arrows).

## Discussion

Thanks to their super-elasticity and shape memory, the NiTi alloys fit to root canal preparation better than the stainless alloys do.^17^ Because of the heat and stress that they are exposed to, NiTi alloys exhibit a phase transition between two crystal structures named austenite and martensite.^18^ At a temperature higher than the transformation temperature, the NiTi alloy is mainly in austenite phase and exhibits super-elastic properties, whereas their hardness reduces and elasticity increases at lower temperatures.^19^ The aim of the present study was to compare the cyclic fatigue resistance of the 2Shape, ESX and TF files at intracanal temperature (35°C). Currently, most NiTi files are manufactured with thermo-mechanically treated alloys. It has been demonstrated that the cyclic fatigue resistances of thermo-mechanically treated NiTi file is affected by the ambient temperature.^16,20,21^ Thus, in the present study, all the NiTi files were tested in a water bath at 35°C in order to mimic the clinical conditions.

In cyclic fatigue tests, the diameter of file at its maximum curvature point within an artificial canal has affected its cyclic fatigue resistance.^22^ In the present study, the apical diameters and tapers of the files were selected to be same (25/.04). To ensure that the diameters of the files at the maximum curvature point of the artificial canals were similar, the D5 points of the files were located at the center of the curvature. Moreover, each artificial canal was specifically designed for each instrument in terms of size and taper, giving it a precise trajectory.^23^

According to the results of present study, 2Shape exhibited significantly higher cyclic fatigue resistance than the other groups at 35°C (P<0.05). Thus, the null hypothesis of the present study was refuted. Dosanjh et al^24^ examined the cyclic fatigue resistance of EdgeFile (EdgeEndo, Albuquerque, USA), Vortex Blue (Dentsply Sirona, Baillagues, Switzerland) and ESX files at different temperatures (3°C, 22°C, 37°C and 60°C). They reported that the ESX file had significantly lower cyclic fatigue resistance at all the temperatures. It was reported that the cyclic fatigue resistance of all the files decreased with the increasing temperature, and that the cyclic fatigue resistance of ESX did not significantly decrease between 37°C and 60°C. As the reason for this finding, the authors stated that the file was mainly in austenite phase at the boy temperature and it was not remarkably affected by the increase in temperature.

In the literature, there are many cyclic fatigue resistance studies carried out using TF.^25,26^ However, since all these studies were carried out at the room temperature, they cannot be directly compared to the present study. In those studies, the cyclic fatigue resistance of TF file was generally found to be higher than files manufactured from M-Wire alloy produced with conventional grinding method.^25,27^ Larsen et al^27^ compared the cyclic fatigue resistance of TF, ProFile GT Series X (Dentsply Sirona), EndoSequence (Brasseler) and ProFile (Dentsply Sirona) files and reported that the resistance of TF file was higher than that of EndoSequence. Similarly, in the present study, TF file exhibited significantly better cyclic fatigue resistance than ESX file, which is a form of EndoSequence file treated with electro-polishing. In parallel with Dosanjh et al,^24^ the authors of the present study believe that this is because ESX file has a harder structure at 35°C when compared to other files that were tested.

Rodrigues CV et al^28^ compared the cyclic fatigue resistance of TF file and RaCe (FKG Dentaire, La Chaux-de-Fonds, Switzerland) files and reported that the cyclic fatigue resistance of TF files was higher than that of RaCe file. They claimed that this is because TF file is manufactured using twisting technology after exposing the metal alloy to a different heat treatment (R-phase).

Since no study is available on 2Shape file in the literature, the findings of the present study cannot be directly compared to those of other studies. In the present study, as the reason for higher cyclic fatigue resistance of 2Shape file when compared to TF and ESX files, it is believed that T-Wire heat treatment used in the production of 2Shape files might have contributed to the increase in file’s elasticity. Higher austenite finish (A_f_) temperature of T-Wire alloy might enable 2Shape files to have softer structure at test temperature (35°C). This might be the reason for higher fracture resistance of 2Shape files when compared to TF and ESX files exposed to different heat treatments.

The cyclic fatigue resistance of files might be affected by not only the alloy characteristics but also by various factors.^29^ The speed rates, at which the files are used, might affect their cyclic fatigue resistance.^30,31^ Lopes et al^31^ exposed the ProTaper Universal F3 and F4 (Maillefer SA, Ballaigues, Switzerland) files to cyclic fatigue test at 300 rpm and 600 rpm rotation speeds and reported that the increase in rotation speed decreased the cyclic fatigue resistance. The authors reported that this might be because the temperature of file surface increases with an increase in rotation speed, causing a thermomechanical stress on the file and decreasing the cyclic fatigue resistance. In the present study, in parallel with the recommendations of manufacturers, ESX and TF files were used at 500 rpm speed and 2Shape file was used at 300 rpm. The authors of the present study believe that the rotation speed of 2Shape file might play a role in its higher cyclic fatigue resistance when compared to ESX and TF.

## Conclusion

Within the limitations of the present study, it was determined that the cyclic fatigue resistance of 2Shape files at the intracanal temperature was higher than TF and ESX files. It is believed that metallurgical analysis of 2Shape might contribute to a better understanding of the cyclic fatigue resistance of these files.

## Author Contributions

Conception - K.Y.; Design – G.U.; Supervision –T.Ö.; Data Collection and/or Processing - G.U., I.I., I.I.P.; Analysis and/or Interpretation - K.Y; Literature Review - G.U., I.I., I.I.P.; Writer – G.U., I.I., I.I.P.; Critical Review - G.U., I.I., I.I.P. All authors have read and approved the final manuscript.

## Acknowledgements

None.

## Competing interests

The authors declare no competing interests with regards to the authorship and/or publication of this article.

## Ethics approval

Not applicable.
